# Outcome of arthroscopic treatment for symptomatic femoroacetabular impingement

**DOI:** 10.1186/1471-2474-15-394

**Published:** 2014-11-23

**Authors:** Torsten Grønbech Nielsen, Lene Lindberg Miller, Bent Lund, Svend Erik Christiansen, Martin Lind

**Affiliations:** Division of Sports Trauma, Orthopedic Department, Aarhus University Hospital, Tage-Hansens Gade 2, Aaxrhus C, 8000 Aarhus Denmark

**Keywords:** Hip, Arthroscopy, Femoroacetabular impingement, FAI, Labral tear

## Abstract

**Background:**

Recently, arthroscopic-based treatment for hip-related pain with radiological findings of femoroacetabular impingement and labral lesions has been developed.

We aim to present clinical outcome in a single centre patient cohort of patients treated arthroscopically for hip-related pain due to femoroacetabular impingement.

**Methods:**

A total of 117 consecutive patients operated in 2009–2011 were included in this prospective case series (41% male; mean age 37 years; (range 15–70). The indication for arthroscopic treatment of hip-related pain was mechanical hip symptoms and radiological findings of femoroacetabular impingement.

To evaluate hip function and pain level at 1-year and 2–5 years follow up (FU) mHHS (Modified Harris Hip Score), HOS (Hip Outcome Score) and a Numeric Rating Scale (NRS) pain score were used.

**Results:**

Labral tears were seen in 91% of the hip arthroscopies. Cartilage lesions (ICRS grade 2 and above) were seen at the acetabular and femoral articular surfaces in 79% and 15% of cases, respectively. The therapeutic procedures were in 99% of the arthroscopies osteochondroplasty and/or acetabular rim-trimming. In 77% of procedures labral reattachment was performed. The patient evaluated outcome demonstrated significant increases in mHHS and HOS at 1-year follow up and at final FU compared to preoperatively (1 yr: mHHS: 72.1 to 85.3, HOS: 71.4 to 85.1; final FU: mHHS: 72.1 to 83.8, HOS: 71.4 to 83.7). Pain levels decreased significantly from preoperatively to follow ups. Five patients underwent total hip replacement within the follow up period after hip arthroscopy.

**Conclusions:**

Arthroscopic treatment of femoroacetabular impingement improves patient evaluated outcomes. Further studies are needed to determine failure rates and risk factors.

## Background

Over the last decade, a new arthroscopic treatment strategy for femoroacetabular impingement (FAI) and labral tears has been developed [1–7]. FAI was described by Ganz in 2003 as abnormal contact between the femoral head and the acetabular rim [8]. Impingement in the hip was described 100 years ago by Vulpius and Stöffel [9]. There is an increasing scientific evidence that arthroscopic treatment of FAI can give favourable outcome (Table 1).

**Table 1:** **Characteristics of included studies**

Study Year	Year	Male	Age	Sample size	Follow up	PROM	Pre	Post
Larson and Giveans [6]	2008	56%	34.7	96	9.9 month	VAS	6.74	1.88
Horisberger et al. [4]	2010	73%	40.9	105	2.3 years	VAS	5.5	1.5
Larson and Giveans [10]	2009	61% (debrid)	32	44	44 months	VAS	6.5	1.7
		58% (refix)	28	50	41 months	VAS	5.7	0.7
Palmer et. al [5]	2012	49%	40.2	201	46 months	VAS	6.8	2.7
Bardakos et al. [11]*	2008	58%	33	24	1 year	mHHS	65	91
Byrd et al. [12]	2009	69%	33	200	16 months	mHHS	65	85
Clohisy et al. [7]	2010	80%	34	35	2.2 year	mHHS	63.8	85.9
Haviv et al. [13]	2010	80%	37	166	22 months	mHHS	70.7	86.1
Byrd and Jones [9]	2011	67%	34	100	2 years	mHHS	65	86.5
Byrd et al. [14]	2011	74%	28.6	116	19 Months	mHHS	72	96
Philippon et al. [15]	2009	45%	40.6	112	2.3 years	mHHS	58	84.3
						HOS	70	87.8
Nho et al. [2]	2011	72%	22.8	47	27 months	mHHS	68.6	88.5
						HOS	78.8	91.4

*MHHS is multiplied with 1.1. This makes the scores comparable with the other studies [16].

Initially, FAI was treated surgically with open dislocation of the hip as described by Ganz [8]. In the last decade it has become more common with an arthroscopic treatment strategy. The disadvantage of open and more invasive operation is a slower recovery due to more extensive soft tissue damage and the need of screw removal in the greater trochanter because of persistent bursitis [4, 17]. To avoid those complications, arthroscopic treatment of FAI have become increasingly popular especially during the last five years. This less invasive surgery leads to a faster rehabilitation and less restrictions [1–3, 6, 7, 17].

Different morphological features such as CAM-type, Pincer-type and Mixed types leads to abnormal contact between the bones in the hip joint and subsequently labral and cartilage lesions. Finally these changes can lead to arthritis [8].

*CAM-type* is normally seen in younger athletic males and is recognised as an abnormal femoral head-neck junction with excessive bone apposition.[1] This bony bump can result in collision with the anterosuperior acetabulum subsequently leading to labral lesions and chondral damage adjacent to the labral detachment [7, 8, 18–20].

*Pincer-type* is more common in middle aged women [1]. Abnormalities are seen as partial overcoverage of the acetabular wall or more generally overcoverage (Coxa profunda). Linear contact between the acetabular rim and the femoral head junctions leads to labral and chondral damage [7, 8, 18–20].

*Mixed-type* is a combination of both CAM and Pincer bony pathology [6, 9, 17, 19, 20].

Although there are different surgical techniques for treatment of FAI, there is no consensus about the optimal patient related outcome measure (PROM) to evaluate clinical outcome after surgical treatment.

Hetaimish et al. evaluated the consistency of the reporting of both clinical and radiographic outcome of FAI. They included 29 eligible studies and found that mHHS, HOS and VAS were used in 45%, 24% and 10% of the articles, respectively. Other PROMS such as NAHS, patient satisfaction and WOMAC were used in 28%, 28% and 14%, respectively [21].

Table 1 compares outcome scores from different FAI studies with this present study.

The purpose of the present study is to present intra operative findings and clinical outcome scores for patients treated arthroscopically for hip-related pain suspected to be due to femoroacetabular impingement. We hypothesised that arthroscopic osteochondroplasty treatment for FAI reduced hip pain and symptoms and improved hip function.

## Methods

We included 117 consecutive patients treated arthroscopically for symptomatic FAI. Patients were treated from 2009 to 2011 and followed prospectively. The study was improved by The Central Denmark Region Committees on Health and Research Ethics (1-10-72-219-14). According to The Central Denmark Region Committees on Health and Research Ethics patient informed consent is not required.

Indications for arthroscopy were mechanical hip symptoms and radiological findings of FAI. The mechanical symptoms were; hip pain, restricted hip motion and/or positive FAI Impingement test.

CAM deformity was defined as a nonspherical femoral head engaging against the articular surface of the acetabulum. Pincer deformity was defined as an overhang of the anterolaterale rim of the acetabulum. Radiographical definition of CAM deformity was alpha angle >55°, and pincer deformity was center Edge (CE) angle >35° and/or cross-over sign [22].

Intraarticular findings were described for cartilage status on the femoral head and acetabular joint surfaces using International Cartilage Repair Society (ICRS) grading [23]. All surgical treatments were performed by two experienced surgeons.

The patient material consisted of 41% men with a mean age of 37 years (range 15–70 years). One-year postoperative data collection was performed at average 13 months after surgery (range 8–22). Four patients were lost to follow up; one of these had a total hip replacement within the first year. Final FU postoperative data was collected at average 40 months (range 24–60). 75% of patients completed final FU.

### Surgical technique

All hip arthroscopies were performed in general anesthesia with patients in supine position. The patients were placed on a fracture table and at the beginning of the procedure trial traction was performed to see if the hip could be distracted properly. Procedures were performed by introducing a fluoroscopy-guided spinal needle in the central compartment from the anterolateral portal. A second portal was established through a mid-anterior portal under direct vision. At both portals a capsulotomy was done to help movement of the instruments in the joint. A diagnostic round was then performed in the joint and all pathologies were registered and subsequently surgery in the central compartment was performed. If a labral tear was noted or damaged to the chondro-labral junction, a debridement of the acetabular rim was done and part of the bony rim was taken down and the labrum reattached with suture anchors. Cartilage damage was debrided and if grade IV damage was found, a microfracture was performed (lesions <2 cm^2^).

After completing the central compartment procedures traction was released and the hip was flexed to approximately 45° and the arthroscope moved to the peripheral compartment to evaluate CAM impingement. Then a cheilectomy was performed from the medial synovial fold to the lateral synovial fold. The cheilectomy was done under direct vision and the hip was moved from flexion to full extension and rotated to check for correct resection of the bump.

At the end of the procedure a pain catheter was placed in the peripheral compartment and local analgesics were administered for two days postoperatively. The patients were guided in removing the pain catheter afterwards.

### Outcome measurements

The following patient reported outcome scores (PROMs) were used for determining clinical outcome: mHHS [16] (0–100 with 100 as the optimal result), HOS [24] (0–100 with 100 as the optimal result) and Numeric Rating Scale (NRS) - pain score [25] (0–10 with 0 as the optimal result). Data were collected preoperatively, at one year follow up and at final follow up between 2–5 years.

### Exclusion criteria

Patients with FAI deformities and symptoms after Peri Acetebular Osteotomy (PAO) or bony deformity after Calve legg Perthe’s Disease were excluded from the present patient material.

### Rehabilitation

All patients were treated as outpatients’ procedures. Preoperatively, a physiotherapist instructed the patient in a designated rehabilitation programme including strengthening of hip flexor, adductor, abductor muscle groups, active mobilisation and crutches. Full weight bearing as tolerated with crutches was allowed from day one and most patients used crutches 2–4 weeks postoperatively. Patients with labral refixation were not allowed to abduct >25° and external rotate >20° for the first three postoperative weeks. Patients were allowed graduately to resume sports activities three months postoperatively. Sports with pivoting were not allowed until 6–8 months postoperatively.

### Statistical analysis

Difference between preoperative and postoperative PROM values were analysed using the Student t-test. *P* values below 0.05 were considered to be statistically significant. Comparisons were made related to age, sex, cartilage damage, total traction time, labral treatment and positive post operative FAI Impingement test.

## Results

Radiological findings of isolated CAM deformities were seen in only one patient, seven patients had isolated pincer deformities and the remaining 109 patients had mixed type FAI deformities.

Pre operative radiological findings are listed in Table 2.

**Table 2:** **Radiology findings**

Parameter	Mean ± SD
Alpha angle (grading)	75.6 ± 9.8
CE angle (grading)	32.1 ± 6.8
Joint space width (JSW) (mm)	3.6 ± 0.6

Intraoperative acetabular cartilage status was evaluated according to the ICRS classification; 7% were grade 0, 15% were grade 1, 39% were grade 2, 28%, were grade 3 and 11% were grade 4. Intraoperative labral lesions were observed in 91% of cases. In 77% of cases a labral reattachment was performed. CAM deformity resection was performed in 94% and pincer deformity resection was performed in 99% of the cases.

### Clinical outcome

Significant improvements from preoperative status to FU were found for all PROMs (Table 3). A clinically relevant improvement of 10 points or more was seen in 51.2% and 50.0% for mHHS and HOS, respectively. A 2-point improvement on the NRS pain score was seen in 48.3% of patients.

**Table 3:** **Comparisons of PROMs at baseline and FU**

	Preoperatively	Postoperatively FU	*P* Value
	Mean ± SD	Mean ± SD	
**All data (117 ptt)**			
mHHS	72.1 ± 16.8	83.1 ± 16.9	< 0.001
HOS	71.4 ± 17.0	83.4 ± 17.4	< 0.001
NRS	5.0 ± 2.7	3.7 ± 2.9	< 0.001
**AGE >40 (52 ptt)**			
mHHS	71.6 ± 17.0	77.9 ± 19,7	0.11
HOS	68.7 ± 18.7	77.3 ± 19.8	0.048
NRS	5.2 ± 3.0	4.2 ± 3.2	0.07
**ICRS > grade 2 (49 ptt)**			
mHHS	74.6 ± 16.0	84.0 ± 13.6	< 0.01
HOS	73.5 ± 16.9	85.3 ± 15.0	< 0.01
NRS	4.4 ± 2.6	3.7 ± 2.8	0.14

A total of 38.9% and 47.4 of the patients with an ICRS Cartilage damage > grade 2 (50 patients) improved >10 points by HOS and mHHS scores respectively, NRS was improved in 57.9% of these patients.

No significant differences in FU PROM values were seen between patients with ICRS >2 cartilage injury compared to the group with ICRS <2 (mHHS: p = 0.66, HOS: p = 0.38, NRS: p = 0.88).

A significant difference in FU PROM values comparing age groups >40 years and <40 years was seen but with no difference in pain scores (mHHS: p = 0.02, HOS: p = 0.01, NRS: p = 0.25).

No significant differences in one-year PROM values were seen between patients having a labral refixation or labral resection. (mHHS: p = 0.60, HOS: p = 0.28, NRS: p = 0.72).

Mean traction time during surgery was 50.4 ± 21.3 minutes (median =45). Traction time shorter or longer than 45 minutes did not significantly affect outcome measures.

### Failures and reoperations

Failure rate based on subjective outcome in the present study was 19.5% and 9.5% based on a 10-point drop of mHHS and HOS, respectively from preoperative to FU. Based on the pain score, definition of failure, with an 1 point increase, at 1 year follow up were 36.0%. Five patients were reoperated with total hip replacement (THR). Two other patients were scheduled for THR after the follow up period. These patients (mean age 48) all had a cartilage injuries ICRS grade 4 at the acetabular rim and ICRS grade 2 changes on the femoral head.

## Discussion

This prospective consecutive study demonstrated that patient with symptomatic FAI benefit from a hip arthroscopic procedure involving removal of bone tissue causing FAI and labral procedures leading to significant improvements in mHHS and HOS one year postoperatively. Similarly, a significant fall in pain scores was seen. These results supported our hypothesis of reduced hip pain and symptoms and improved hip function after arthroscopic treatment of FAI. This is the first study to demonstrate that labral fixation as an adjunct to removal of pincer impingement inducing bone at the acetabulum and traction time does not affect subjective outcome.

When comparing the outcome scores of the present study with the studies listed in Table 1 the results are very similar. Mean mHHS at follow-up was 83.1 compared to 84–96 and mean HOS was postoperatively was 83.4 compared to 88–91. The mean improvement for mHHS was in present study 11.0. Haviv et al. [13] have found a mean improvement at 15 and the other studies listed in Table 1 presented improvements of 20–26 points. As our patient material is similar to the previous studies, we suggest social and cultural factors when responding to the subjective outcome instruments to be an explanation for this discrepancy. In a public healthcare system patient expectations to outcome is potentially different than in a private system. Mean improvement in HOS in our study was 12.0 compared to 12–17 in previously published studies. The NRS pain score decreased with 1.3 points compared to 4.0-5.0 points in other studies (Table 1). The differences in pain score improvement could be explained by the same factor as mentioned above.

Other studies have found a correlation between chondral damage in FAI patients and subjective outcome. Haviv et al. [13] evaluated the impact of cartilage injury on clinical outcome of cartilage damage in FAI patients. They found no difference in improvement of mHHS improvement between different degrees of cartilage injury. However at final follow-up limited cartilage injury resulted in better outcome than when significant cartilage injury was present [13]. Philippon et al. found that poor cartilage status lead to a poor subjective outcome. At 2.3 years follow-up mild, moderate and poor cartilage status had mHHS scores of 87, 79 and 62, respectively [15]. The present study did not find any difference in mHHS outcome between cartilage injuries ICRS grades 1–2 (69 patients) and grades 3–4 (48 patients). The mHHS were 82.5 and 85.0, respectively. However, all failures that had THR reoperation had severe cartilage injuries grade IV.

A total of 78% of the patients had a labral refixation after removal of acetabular bone tissue. The patients with labral refixation did not have poorer subjective outcome than patients without this procedure. Larson and Giveans [10] demonstrated that labrum refixation leads to improved subjective outcome compared to labral resection in two patient cohorts with labral damage. Another prospective randomised study by Krych et al. [26] showed the same improvement of outcome scores.

Labral lesions combined with pincer impingement are typically treated with resection of the acetabular rim. Thus, patients requiring a release of the labrum because of pincer impingement had a more pronounced FAI pathology compared with patients with more limited pincer deformity.

Significantly lower mHHS and HOS were found related to age. Patients >40 years scored significantly lower than patients <40 years. When comparing the older and the younger patient group no difference in cartilage damage was found. Cartilage injury ICRS >2, was 78% in both groups. However, significantly lower joint space width (JSW) was found in patients >40 years compared to patients <40 years (p = 0.002).

The Alpha angle for symptomatic FAI patients in other studies ranged from 61.3° – 80.0° [2, 4, 5, 7, 15, 27]. This correlates well with the findings of 75.6° in the present study.

Only one of the studies used for comparison with the present study has used CE-angle for radiological evaluation. Nho et al. found a CE-angle at 36.5° compared to the 32.1° found in the present study [2].

The mean JSW in this present study was 3.6 mm. Philippon et al. found 3.4 mm and 3.6 mm in their cohorts [15, 28].

The THR reoperation rate in the present study is similar to other published studies. In 96 patients Larson and Giveans found a 3% THR reoperation rate [6]. All had grade 4 acetabular chondral lesions with delamination of cartilage from the subchondral bone. In the study by Byrd et al. [12] only 1 patient had a THR in 200 patients. The patient had grade 4 articular lesion at both femoral head and the acetabulum. Overall higher age, higher degree of cartilage injury and/or osteoarthritis are predictors for THR reoperations [13, 15, 28, 29].

The present study has several limitations. One of the most important is the lack of control group consisting of non-operated/conservatively treated FAI patients. Evaluation of outcome after hip arthroscopy is also challenged by the lack of dedicated subjective outcome instruments for patients with non-arthritic hip pathology. The instruments used in the present study, mHHS and HOS, both have limitations; mHHS is connected with floor and ceiling effect and HOS is not designed for this specific patient population.

There are several strengths in this present study. This study is a consecutive, prospective case series including a relatively large cohort of 117 patients. The study had excellent completeness with 90% and 75% at 1 year and long time follow up, respectively.

## Conclusion

In conclusion patients with pain related to mechanical hip symptoms and radiological findings of FAI will benefit from hip arthroscopy with resection of CAM and Pincer bony deformities. Their functional level will increase and their pain level will decrease significantly. Further studies are needed to determine failure rates and outcome risk factors.
